# Supplementary material for: Effect of Eco-Friendly Application of Bee Honey Solution on Yield, Physio-Chemical, Antioxidants, and Enzyme Gene Expressions in Excessive Nitrogen-Stressed Common Bean (Phaseolus vulgaris L.) Plants
Source: Plants (Basel). 2023 Sep 29;12(19):3435. doi: 10.3390/plants12193435 (PMC10575117; doi:10.3390/plants12193435)
Supplement: Supplementary file 1 [file plants-12-03435-s001.zip › plants-2603072-supplementary.pdf]

Table S1. Average weather data of the experimental location (Beni Suif District) during the two growing seasons

Month	Day °C	Night °C	ARH (%)	AWS (km h^{-1})	AP (mm d^{-1})
2021 season					
September	34.4	18.1	52.2	17.0	0.41
October	31.5	16.3	59.4	17.2	0.45
November	30.0	14.0	47.9	17.8	0.50
2022 season					
September	34.2	17.5	54.0	17.5	0.48
October	31.0	16.6	59.5	17.5	0.54
November	29.8	13.8	46.2	17.6	0.60

Day °C= Average day temperature, Night °C= Average night temperature, ARH= Average relative humidity, AWS= Average wind speed, and AP= Average precipitation.

Table S2. Physicochemical properties of the soil used in this study

Soil physical characteristics

Depth (cm)	Particle size distribution				ρ_b (g cm^{-3})	K_{sat} (cm h^{-1})	$\theta_{Fc.}$ (%)	$\theta_{W.P.}$ (%)	A.W. (%)
	Sand (%)	Silt (%)	Clay (%)	Texture					
0-30	20.6	37.8	41.6	Clay loamy	1.74	2.28	28.4	12.2	16.4

Soil chemical properties

pH	7.68	N (%)	0.06
ECe (dS m^{-1})	2.34	P (mg kg^{-1} soil)	67.8
CEC (cmole kg^{-1})	13.4	K (mg kg^{-1} soil)	486
$CaCO_3$ (%)	4.68	Fe (mg kg^{-1} soil)	9.84
OM (%)	1.08	Mn (mg kg^{-1} soil)	6.71
ESP	10.4	Zn (mg kg^{-1} soil)	0.96
SAR	8.62	Cu (mg kg^{-1} soil)	0.67

ρ_b, Bulk density; K_{sat}, Hydraulic conductivity; $\theta_{Fc.}$, Volumetric water content at field capacity; $\theta_{W.P.}$, Volumetric water content at wilting point; A.W., Available water; OM, Organic matter; ESP, Exchangeable sodium percentage and SAR, Sodium adsorption ratio.

Table S3. Chemical composition of irrigation water

Concentration of ions (meq L^{-1})								EC (dS m^{-1})	pH	SAR
CO_3^{2-}	HCO_3^-	SO_4^{2-}	Cl^-	Mg^{2+}	Ca^{2+}	K^+	Na^+			
0.00	2.11	3.25	11.7	1.74	5.40	1.39	6.13	1.66	7.44	2.90

EC= Electrical conductivity, and SAR= Sodium adsorption ratio.

Table S4. Primers sequences for RT-PCR of the stress-related genes in wheat.

The gene	Reference Seq.	5′ - 3′ primer sequence	T_A
Actin 1	AB181991	F: CTCTGACAATTTCCCGCTCA<br>R: ACACGCTTCCTCATGCTATCC	58 °C
Actin 2	AT2G37620	F: GCTATTCAAGCCGTGCTTTC<br>R: AGCATGTGGAAGGGCATAAC	
SOD	MG893090.1	F: TTCGCCATGCTGGTGATCTT<br>R: CATGGACAACTACGGCCCTT	
CAT	GU984379	F: GGCTGCTTGAAGTTGTTCTCCT<br>R: CTGCTAGTACCTCCTGATCCGTT	
APX	KU747079.1	F: TGGCCTGCTCTTCCTCTAGT<br>R: CATGCCACGCTAATCGAAGC	
GR	KX828561.1	F: CAACGCGCTTTGGTAACTCC<br>R: GGGCCCTAATGAAGTGGAGG	
